# Down-regulation of CITED2 attenuates breast tumor growth, vessel formation and TGF-β-induced expression of VEGFA

**DOI:** 10.18632/oncotarget.14048

**Published:** 2016-12-21

**Authors:** Swaathi Jayaraman, Michele Doucet, Scott L Kominsky

**Affiliations:** ^1^ Department of Orthopaedic Surgery, Johns Hopkins University School of Medicine, Baltimore, MD, USA

**Keywords:** CITED2, primary tumor growth, vasculature, VEGFA isoforms, TGF-β

## Abstract

While we previously demonstrated that CITED2 expression in primary breast tumor tissues is elevated relative to normal mammary epithelium and inversely correlated with patient survival, its functional impact on primary tumor development and progression remained unknown. To address this issue, we examined the effect of CITED2 silencing on the growth of human breast cancer cell lines MDA-MB-231 and MDA-MB-468 following orthotopic administration *in vivo*. Here, we show that CITED2 silencing significantly attenuated MDA-MB-231 primary tumor growth concordant with reduced tumor vascularization, while MDA-MB-468 primary tumor growth and tumor vascularization remained unaffected. Correspondingly, expression of VEGFA was significantly reduced in shCITED2-expressing MDA-MB-231, but not MDA-MB-468 tumors. Consistent with the observed pattern of vascularization and VEGFA expression, we found that TGF-β stimulation induced expression of VEGFA and enhanced CITED2 recruitment to the VEGFA promoter in MDA-MA-231 cells, while failing to induce VEGFA expression in MDA-MB-468 cells. Further supporting its involvement in TGF-β-induced expression of VEGFA, CITED2 silencing prevented TGF-β induction of VEGFA expression in MDA-MB-231 cells. Collectively, these data indicate that CITED2 regulates primary breast tumor growth, likely by influencing tumor vasculature via TGF-β-dependent regulation of VEGFA.

## INTRODUCTION

After lung cancer, breast cancer is the most commonly diagnosed cancer worldwide and is the second leading cause of cancer deaths in women [1]. In the United States alone, approximately 40,000 women succumb to breast cancer each year, with one in eight women developing breast cancer in their lifetime [2]. While the heterogeneity of the primary tumor makes treatment challenging, it is further compounded by our limited knowledge of the key drivers of breast cancer progression. Identification of these factors and elucidation of their mechanism of action is critical to the development of novel prognostic and treatment modalities for the management of this disease.

Cbp/p300–interacting transactivator with Glu/Asp–rich carboxy-terminal domain-2 (CITED2) is a non-DNA binding transcriptional co-regulator that directly interacts with host of transcription factors (LhX2, TFAP2, Smad2/3, PPARγ, estrogen receptor) and co-activators (p300/CBP) thereby influencing their ability to activate gene transcription [3–7]. CITED2 can also serve as co-repressor where it can negatively regulate HIF1α-mediated gene transcription [8]. Owing to its critical role in development, particularly of the liver, lung, heart and neural tube [9–11], deletion of CITED2 in mice results in embryonic lethality. CITED2 is also important for lens morphogenesis [12]. In addition to its prominent role in development, CITED2 has been implicated in malignancies such as skin, colon, and lung cancer [13–15]. In initial studies, we identified the ability of CITED2 to facilitate bone metastasis in a murine mammary cancer model [16]. Extending this work to breast cancer in humans, we found that CITED2 expression in primary tumor and metastatic tissues was elevated relative to normal mammary epithelium, with CITED2 levels in primary tumors inversely correlating with patient survival [7, 16–17]. While we demonstrated the ability of CITED2 to facilitate metastatic dissemination and colonization of secondary sites [17], its impact on early disease progression remained unknown.

To fill this knowledge gap, we investigated the role of CITED2 in the establishment and progression of breast cancer at the primary site. Utilizing two murine orthotopic models of human breast cancer, we provide evidence that CITED2 regulates primary tumor growth, likely secondary to effects on the formation of tumor vasculature. Further, corresponding with its effects on vascularization, we demonstrate that CITED2 regulates VEGFA expression, at least in part, by modulating its induction by TGF-β. Collectively, these data provide the first evidence of a role for CITED2 in primary breast cancer progression and a potential mechanism for its action.

## RESULTS

### CITED2 silencing attenuates MDA-MB-231, but not MDA-MB-468 tumor growth

We previously reported that CITED2 mRNA and protein expression was significantly elevated in primary breast tumors of breast cancer patients relative to normal mammary epithelium, with CITED2 levels being inversely correlated with patient survival [7, 17]. To explore the functional impact of CITED2 in primary breast cancer, we utilized the human MDA-MB-231 and MDA-MB-468 breast cancer cell lines. These cell lines readily establish tumors following orthotopic administration in mice [18, 19], and as we have shown previously, express elevated levels of CITED2 relative to human mammary epithelial cells [16]. MDA-MB-231 and MDA-MB-468 cells were stably infected with a lentiviral expression vector containing either shRNA specific for CITED2 (shCITED2) or scrambled shRNA (scramble). qRT-PCR and western blot analysis revealed greater than 60% reduction in CITED2 protein expression in both MDA-MB-231 and MDA-MB-468 cells (Supplementary Figure S1), which was consistent with CITED2 reduction levels reported previously [17]. Further, CITED2 silencing in these cells did not affect tumor cell proliferation *in vitro* [17].

**Figure 1:**
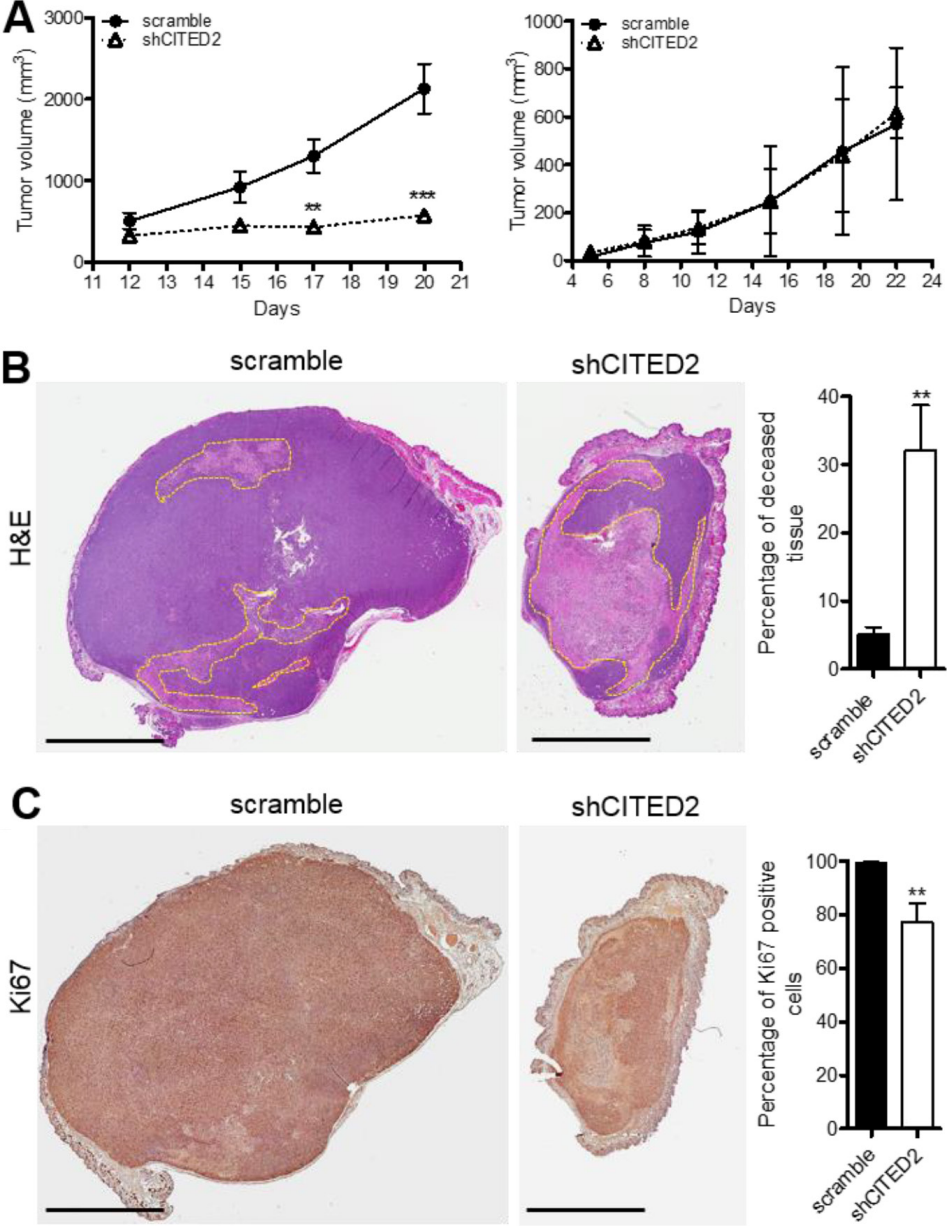
CITED2 silencing attenuates MDA-MB-231, but not MDA-MB-468 tumor growth (**A**) Average tumor volume measured over time following injection of scramble or shCITED2-expressing MDA-MB-231 (left) and MDA-MB-468 (right) cells into the mammary fat pad of athymic nude mice. (**B**) Representative H&E-stained sections of tumor from each experimental group. The pale pink areas highlighted by yellow dotted lines denote deceased tissues. The adjacent histogram represents the average percentage of deceased tissue displayed by the tumors in each experimental group as measured on H&E-stained sections. (**C**) Representative images of immunohistochemical analysis of Ki67 protein expression from each experimental group. Ki67 was identified using DAB (brown) and visualized by light microscopy. The adjacent histogram represents the average percentage of Ki67 positive cells displayed in each experimental group. (**P < 0.01, ***P < 0.001). Scale bar: 4 mm.

MDA-MB-231 and MDA-MB-468 cells were administered into the mammary fat pad of athymic nude mice and tumor growth was measured by caliper. Although mice administered scramble and shCITED2-expressing MDA-MD-231 cells developed tumors that were similar in size at the onset, tumors in the scramble group grew steadily while tumor growth in the shCITED2 group appeared stagnant, resulting in a significantly lower tumor size relative to the scramble group (Figure 1A, left). In contrast, mice administered with scramble and shCITED2-expressing MDA-MD-468 cells developed tumors that displayed nearly identical growth patterns, indicating that CITED2 silencing did not impact the growth of MDA-MB-468 tumors (Figure 1A, right). Histological staining of scramble and shCITED2-expressing MDA-MB-231 tumors revealed that shCITED2-expressing tumors were markedly smaller in size and displayed higher percentage of deceased tissue (32%) relative to those in the scramble group (5%) (Figure 1B). Staining for Ki67, a marker of cellular proliferation, confirmed that the fraction of growing tumor cells was significantly reduced in shCITED2-expressing tumors (72%) relative to scramble-expressing tumors (98%) (Figure 1C). Taken together, these data indicate that CITED2 silencing selectively attenuates growth of MDA-MB-231, but not MDA-MB-468, orthotopic tumors *in vivo*.

### CITED2 silencing attenuates MDA-MB-231, but not MDA-MB-468 tumor vascular area and diameter

**Figure 2:**
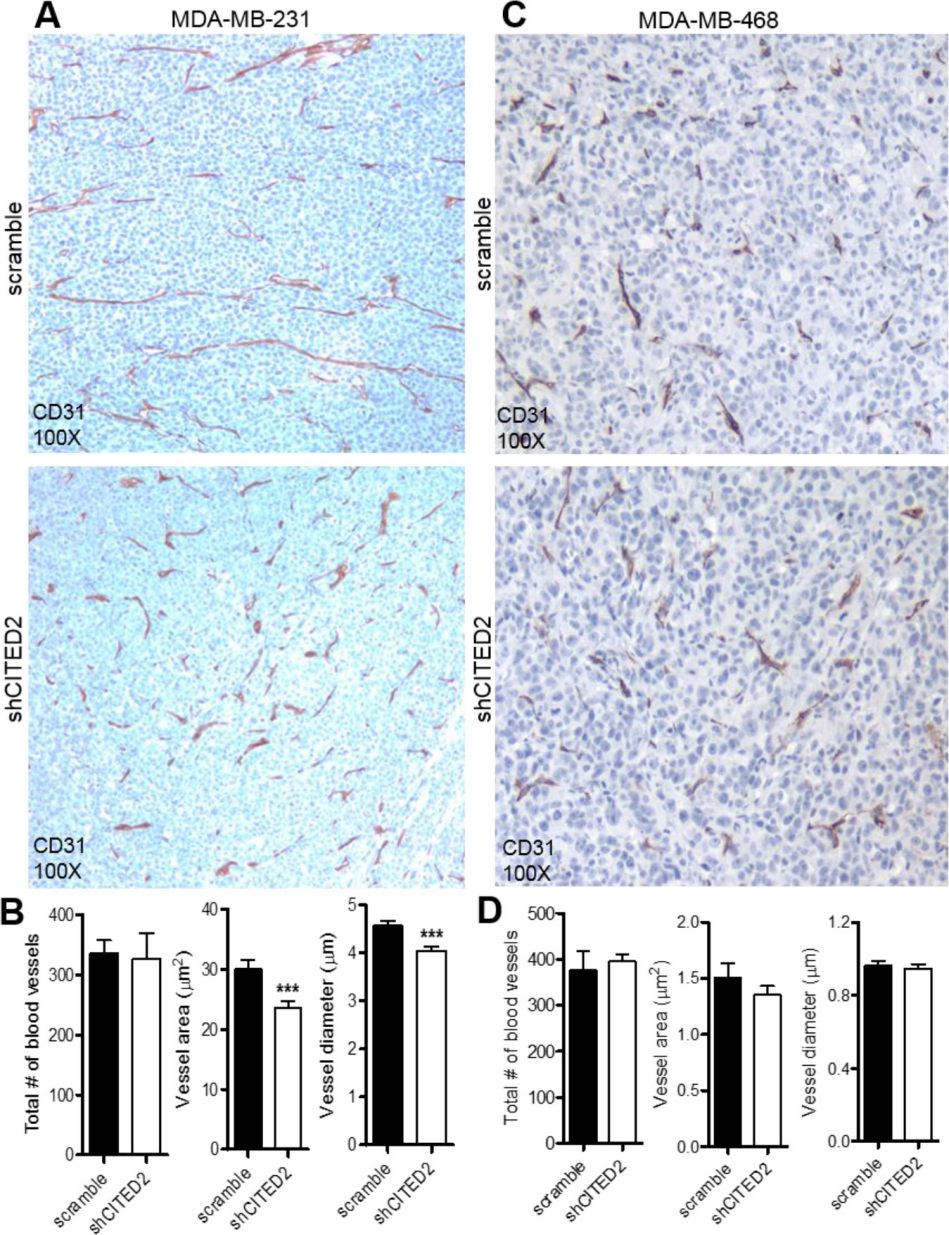
CITED2 silencing attenuates MDA-MB-231, but not MDA-MB-468 tumor vascular area and diameter (**A** and **C**) Immunohistochemical analysis of CD31 protein expression (brown) in scramble and shCITED2-expressing tumors. Magnification: 100X. (**B** and **D**) Histograms representing the average overall number of blood vessels, vessel area and vessel diameter in each experimental group. (***P < 0.001).

The inability of CITED2 silencing to impact MDA-MB-231 cell proliferation *in vitro* suggested that the reduced tumor growth displayed by shCITED2-expressing MDA-MB-231 tumors was due to an indirect effect. While tumors less than 1–2 mm^3^ in size can obtain oxygen and nutrients by simple passive diffusion, growth and survival of tumors beyond 1–2 mm^3^ requires a dedicated blood supply, which is provided by tumor angiogenesis [20]. To determine whether the high incidence of deceased tissues and reduced cellular proliferation observed in shCITED2-expressing MDA-MB-231 tumors was potentially impacted by defects in tumor-induced angiogenesis, scramble and shCITED2-expressing tumors were stained for CD31, a marker of blood vessel lining endothelial cells. Although CITED2 silencing did not affect the total number of blood vessels observed between the two tumor groups, vessels in the shCITED2-expressing tumors appeared shorter (Figure 2A, top and bottom images and Figure 2B, left graph). By ImageJ analysis of vessel features in the healthy regions of the tumor, the average vessel area and diameter were significantly reduced in shCITED2-expressing tumors relative to the scramble group (Figure 2B, center and right graph). In contrast, CITED2 silencing did not affect the vasculature of MDA-MB-468 tumors (Figure 2C–2D). Taken together, these data demonstrate that CITED2 silencing selectively alters MDA-MB-231 but not MDA-MB-468 tumor vessel formation.

### CITED2 silencing attenuates expression of VEGFA isoforms in MDA-MB-231 tumors

Vascular endothelial growth factor A (VEGFA) is widely accepted as the most potent stimulator of tumor angiogenesis [21], with anti-VEGFA therapies revealing promising outcomes in pre-clinical trials [22, 23]. Two families of VEGFA isoforms, generated by alternate splicing, are known to exist [24]. Among the six pro-angiogenic VEGFA isoforms (VEGFA_121_, VEGFA_145_, VEGFA_148_, VEGFA_165_, VEGF_183_, and VEGFA_189_), VEGFA_165_ is the most abundant and frequently expressed, followed by VEGFA_121_. Expression of these VEGFA isoforms has been observed in several cancer types including breast cancer [25–28].

**Figure 3:**
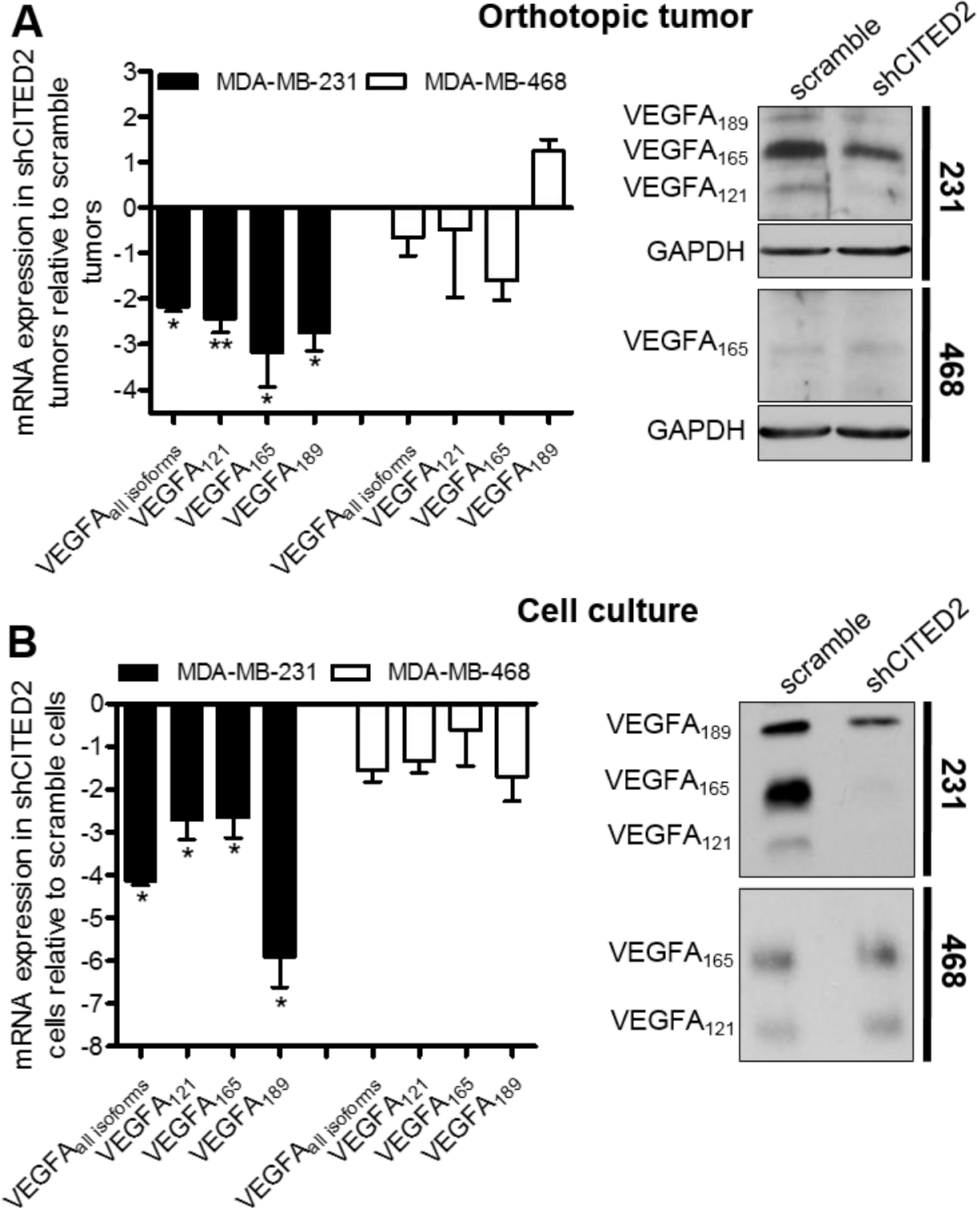
CITED2 silencing attenuates expression of VEGFA isoforms in MDA-MB-231 tumors (**A** and **B**) Left: mRNA expression of VEGFA isoforms in the orthotopic tumors (A) and cells grown *in vitro* (B) as determined by qRT-PCR (*P < 0.05, **P < 0.01). Right: *Top*, Western blot analysis of VEGFA isoforms performed on equal amounts of protein obtained from total cell lysates of orthotopic tumor. GAPDH served as the loading control. *Bottom*, Western blot analysis of VEGFA isoforms performed on equal amounts of protein obtained from serum-free conditioned media of cells grown *in vitro*.

Reports indicate that VEGFA_121_ and VEGFA_165_ influence blood vessel formation, affecting both vessel diameter and length [29]. To determine whether the reduced vessel area and diameter observed in shCITED2-expressing MDA-MB-231 tumors could be due to effects on VEGFA expression, we examined the mRNA and protein expression of VEGFA in scramble and shCITED2-expressing MDA-MB-231 tumors. By qRT-PCR analysis, levels of total VEGFA mRNA were significantly reduced in shCITED2-expressing tumors relative to scramble-expressing tumors (Figure 3A, left). Western blot analysis revealed that MDA-MB-231 tumors expressed VEGFA_121_, VEGFA_165_, and VEGFA_189_ isoforms at the protein level, and concordant with the mRNA data, protein expression of all three isoforms was reduced in the shCITED2-expressing tumors relative to scramble-expressing tumors (Figure 3A, right). Since CITED2 silencing did not affect MDA-MB-468 tumor growth or vessel formation, we also examined VEGFA expression in scramble and shCITED2-expressing MDA-MD-468 tumors. Correspondingly, CITED2 silencing did not affect either VEGFA mRNA or protein expression (Figure 3A). Consistent with the data obtained from the xenograft MDA-MB-231 and MDA-MB-468 tumor tissues, CITED2 silencing reduced protein expression of VEGFA_121_, VEGFA_165_, and VEGFA_189_ isoforms in MDA-MB-231 cells grown *in vitro*, while having no effect on VEGFA isoform expression in MDA-MB-468 cells (Figure 3B). To determine whether pro-angiogenic factors other than VEGFA is also involved in CITED2 angiogenic effects in MDA-MB-231 tumors, we also examined the expression of Interleukin 6 (IL-6), Fibroblast growth factor 2 (FGF2), Angeopoietin-1 (ANGPT1) and Semaphorin 3C (SEMA3C), all of which are known to play a prominent role in tumor angiogenesis [30–33]. CITED2-silencing did not alter the mRNA expression of these factors, ruling out their potential involvement in CITED2-mediated effects (Supplementary Figure S2).

### CITED2 regulates TGF-β-induced expression of VEGFA

**Figure 4:**
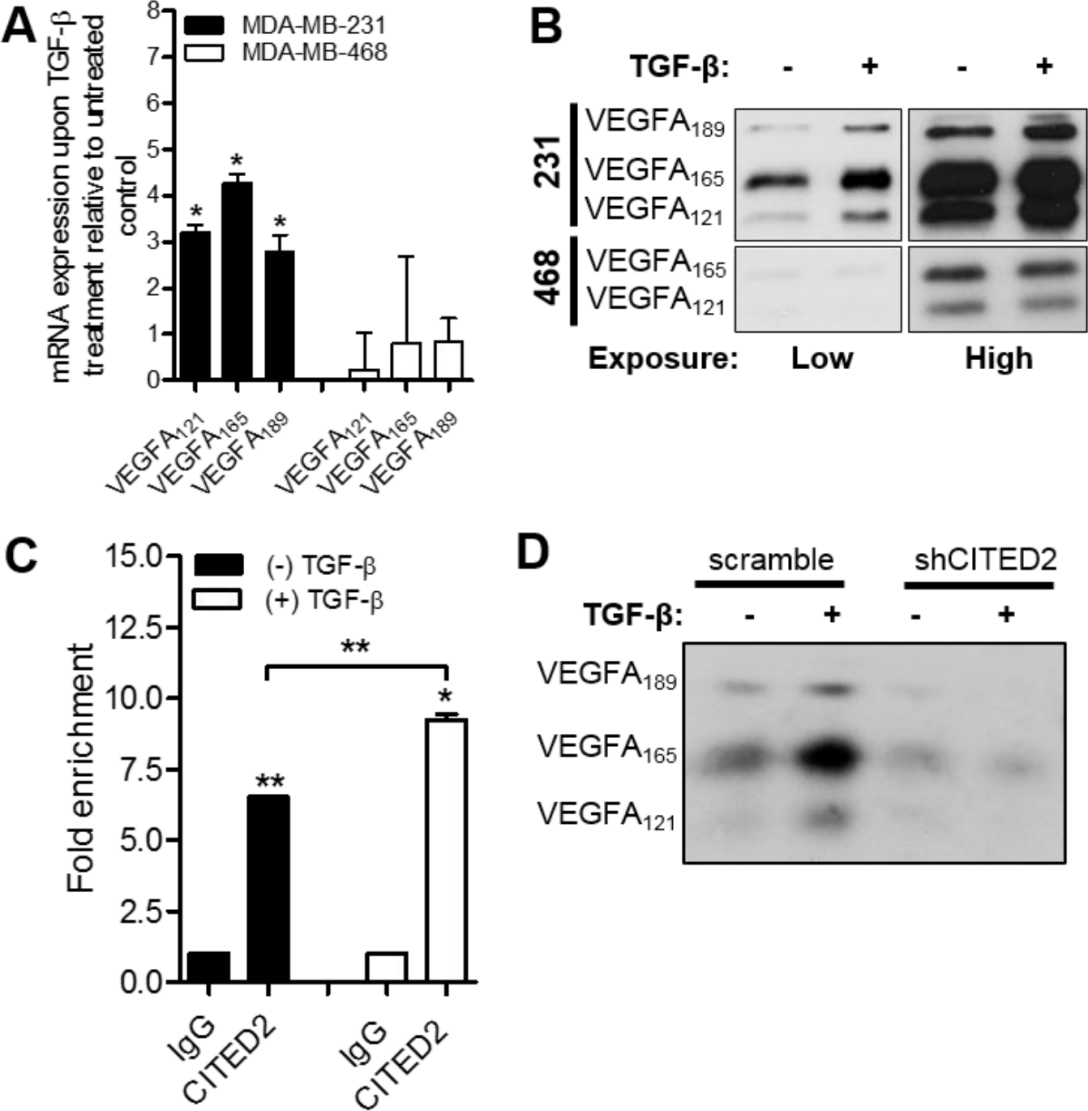
CITED2 regulates TGF-β-induced expression of VEGFA (**A**–**B**) Wild-type MDA-MB-231 and MDA-MB-468 cells were treated with or without 2.5 ng/ml TGF-β for 24 hours. (A) mRNA expression of VEGFA was determined by qRT-PCR. (B) Protein expression of VEGFA was determined by western blot analysis performed on equal amounts of protein obtained from serum-free conditioned media. Protein bands obtained from low (5 seconds) and high (20 seconds) exposure times are presented. (**C**) Localization of CITED2 to the VEGFA promoter as assessed by ChIP assay using anti-CITED2 or IgG antibodies (control) in wild-type MDA-MB-231 cells upon treatment with or without 2.5 ng/ml of TGF-β for 24 hours. (**D**) scramble and shCITED2-expressing MDA-MB-231 cells were treated with or without 2.5 ng/ml TGF-β for 24 hours and western blot analysis of VEGFA isoform expression was performed on equal amounts of protein obtained from serum-free conditioned media. (*P < 0.05, **P < 0.01).

One of the mechanisms responsible for VEGFA expression in both physiologically normal and cancer cells is transforming growth factor-β (TGF-β) stimulation [34–36]. While canonical TGF-β signaling is active in MDA-MB-231 cells [37], it is inhibited in MDA-MB-468 cells due to heterozygous deletion of Smad4 [38]. Since CITED2 has been shown to interact with Smad2/3 and modulate canonical TGF-β signaling in MDA-MB-231 cells [5], we sought to determine whether the differing ability of shCITED2 to inhibit VEGFA expression in MDA-MB-231 versus MDA-MB-468 cells could be due to differences in TGF-β signaling in these cell lines. To test this possibility, we first examined VEGFA expression in both MDA-MB-231 and MDA-MB-468 cells upon TGF-β treatment. While TGF-β induced expression of VEGFA_121_, VEGFA_165_, and VEGFA_189_ at both the mRNA and protein levels in MDA-MB-231 cells, it failed to induce expression of VEGFA isoforms in MDA-MB-468 cells (Figure 4A–4B), consistent with the inability of CITED2 silencing to impact VEGFA expression in this cell line (Figure 3).

Given its role as a transcriptional co-regulator and Smad2/3 binding partner, we next examined whether CITED2 localized to the VEGFA promoter. ChIP analysis in MDA-MB-231 cells revealed that CITED2 localized to the VEGFA promoter (Figure 4C, left), indicating a potential role for CITED2 in the regulation of gene transcription. Furthermore, CITED2 recruitment to the VEGFA promoter was significantly increased upon stimulation with exogenous TGF-β (Figure 4C, right), supporting the involvement of CITED2 in TGF-β-induced activation of VEGFA transcription. Investigating further, we determined the effect of CITED2 silencing on the ability of TGF-β to induce VEGFA expression. While addition of TGF-β to scramble-expressing cells increased expression of VEGFA_121_, VEGFA_165_, and VEGFA_189_ isoforms relative to untreated cells, TGF-β stimulation failed to induce VEGFA expression in shCITED2-expressing cells (Figure 4D). Collectively, these data indicate that CITED2 is a key regulator of TGF-β-induced expression of VEGFA.

## DISCUSSION

Despite improvements in early detection, breast cancer continues to pose a significant challenge. Identification of key factors mediating the establishment and progression of breast cancer is critical for the development of effective treatment modalities to combat this disease. In previous studies, we identified CITED2 as being over-expressed in primary human breast cancers relative to normal mammary epithelium, negatively correlating with survival. Here, we have shown that silencing of the non-DNA binding transcriptional co-regulator CITED2 significantly attenuates the growth of human breast cancer in an orthotopic tumor model, likely secondary to effects on tumor vascularization. Correspondingly, we demonstrate that CITED2 regulates VEGFA expression in breast cancer cells, at least in part via modulation of TGF-β-induced transcription. These data provide a novel role for CITED2 in primary breast cancer and a plausible mechanism for its effects.

Given the multiple mechanisms regulating VEGFA expression, beyond the influence of CITED2 on TGF-β signaling, we also examined its impact on the expression of hypoxia-inducible factor-1α (HIF-1α), a well-known transcriptional regulator of VEGFA [39–41]. CITED2 silencing did not affect either the mRNA or protein expression of HIF-1α in MDA-MB-231 cells grown in cell culture or orthotopically in mice (Supplementary Figure S3), indicating a lack of involvement in CITED2-mediated regulation of VEGFA expression. Also of note, the transcriptional activity of HIF-1α is reportedly repressed by CITED2 via competitive binding to p300/CBP [8], and has been shown to result in decreased VEGFA expression in nucleus pulposus cells [42]. Given this relationship, one might expect that CITED2 silencing in MDA-MB-231 tumors would result in transcriptional activation of the HIF-1α target VEGFA, however the opposite effect was observed. This result indicates that negative regulation of HIF-1α by CITED2 may be a cell type-dependent effect or somehow impaired in breast cancer cells.

The ability of CITED2 to influence both primary breast cancer growth and metastatic progression in model systems [17], underscores its potential importance as a tumor-promoting factor. Since the MDA-MB-231 and MDA-MB-468 cell lines are both representative of the basal subtype of breast cancer, it will be interesting to examine the pro-tumorigenic and pro-metastatic effects of CITED2 in other breast cancer subtypes. Additionally, as a non-DNA-binding transcriptional co-regulator, the biological effects of CITED2 are influenced by the host of transcription factors in the cell. This is well illustrated by the differing effects of CITED2 silencing on TGF-β-mediated VEGFA expression in MDA-MB-231 cells versus MDA-MB-468 cells, which are Smad4-deficient. Thus, it will be important to identify CITED2 binding partners and consider their expression alongside that of CITED2 in order to gauge its potential impact in a given tumor. Such studies will not only further our understanding of CITED2 effects in breast cancer, but may inform its utility in prognostic and therapeutic approaches.

## MATERIALS AND METHODS

### Cell lines and transfection

The human breast cancer cell lines MDA-MB-231 and MDA-MB-468 were obtained from American Type Culture Collection, Rockville, MD (2014) and were cultured as previously described [17]. The cell lines were authenticated by the cell bank using DNA profiling and cytogenetic analysis, and utilized for experiments within six months from the time of resuscitation. Both scramble and shCITED2-expressing MDA-MB-231 and MDA-MB-468 cells were generated as previously described [17]. Briefly, cells were infected with the lentiviral shRNA expression vector pLKO.1-puro (Addgene plasmid 8453) containing siRNA sequence specific for scrambled or CITED2 [5, 43]. For TGF-β treatment, wild-type and shRNA transfected cells were treated with 2.5 ng/ml TGF-β (PeproTech) for 24 hours.

### *In vivo* orthotopic tumor model

All animal experiments were carried out in accordance with the National Research Council's ‘‘Guide to the Care and Use of Laboratory Animals’’. Animal use was approved by the Johns Hopkins Animal Care and Use Committee, animal welfare assurance #A3272-01, protocol #MO10M450.

Tumor cells (1.5 × 10^6^) in 0.1 ml of Hanks’ Balanced Salt Solution (HBSS, Gibco) were injected bilaterally into the third mammary fat pad of five-week-old athymic nude mice (Taconic) [*n* = 5 per group]. Mice were anesthetized by intraperitoneal injection of 0.25 ml of ketamine hydrochloride (100 mg/ml, Hospira) prepared in xylazine solution (2 mg/ml, AnaSed) prior to tumor inoculation. With the onset of tumor growth, the tumor length and width was measured every 2–3 days by external calipers and the tumor volume calculated using the formula: *(Width*^2^ * *Length)/2*. Once a tumor reached the maximum allowable tumor size, all mice were euthanized and the tumors excised. One portion of the tumor was formalin fixed, paraffin embedded, sectioned and subjected to histological and immunohistochemical staining while the other portion was processed for RNA and protein extractions as described below.

### Histological analysis

H&E staining of the scramble and shCITED2 MDA-MB-231 tumor tissues were carried out at the Pathology Department, Johns Hopkins University School of Medicine. The percentage of deceased tissue between the experimental groups was analyzed using the Aperio Imagescope image analysis software (Aperio Technologies).

For Ki67 staining, tissue sections were deparaffinized in xylene (Fisher Scientific), rehydrated through a graded series of ethanol (Pharmco-AAPER) and washed in phosphate buffer saline (PBS, Gibco). Sections were immersed in antigen retrieval solution (Dako) and heated in a steamer for 20 minutes. Cooled sections were washed with PBS and endogenous peroxidase activity was quenched by immersing in 3% hydrogen peroxide (Fischer Scientific) for 10 minutes. Endogenous biotin was then blocked using an Avidin and Biotin blocking kit (DAKO) for 10 minutes and incubated at 37°C for one hour with rabbit anti-human ki67 antibody (1:20 dilution, NB500-0170, Novus Biologicals). PBS-Tween washed sections were incubated with Envision+Dual Link System-HRP (K4063, DAKO) for 30 minutes. Proteins were visualized by addition of the chromogen 3, 3-diaminobenzamindine (DAB; Open Biosystems) and counterstained with hematoxylin Gill No. 3 (Sigma-Aldrich). The percentage of Ki67-positive cells between the experimental groups was analyzed using Aperio Imagescope image analysis software.

For CD31 staining, deparaffinized and dehydrated tissues sections were subjected to antigen retrieval, PBS wash and endogenous peroxidase quenching as describe above. Sections were incubated with protein blocking solution (DAKO) for 20 minutes at room temperature followed by incubation at 4°C for 18 hours with rat anti-mouse CD31 antibody (1:30 dilution; DIA310, Dianova). PBS-washed sections were sequentially incubated for 30 minutes at room temperature with rabbit anti-rat-avidin antibody and streptavidin-horse radish peroxidase (HRP) from the ABC Vector kit (Vector Laboratories Inc.). Proteins were visualized as described above.

### ImageJ analysis of vessel features

Five micron deep tissue sections from each experimental group (*n* = 10 per group) that were stained for CD31 were analyzed for vessel features. The blood vessel area, diameter and total number of blood vessels between the CD31-stained scramble and shCITED2 tumors were analyzed using ImageJ software (National Institute of Health, Bethesda, MD). Briefly, images with set scale bar were converted to greyscale. Upon setting the threshold, all the blood vessels within the image were selected and analyzed for vessel area, radius and total number of blood vessels. The vessel diameter was calculated from the vessel radius.

### Quantitative (q)RT-PCR

Total RNA from cell lines (three biological replicates per experimental condition) were obtained using high pure RNA isolation kit (Roche) and converted to cDNA using a cDNA synthesis kit (Quanta Biosciences) based on manufacturer's instructions. Total RNA from tumor tissues (six tumors per experimental group for MDA-MB-231 and four tumors per experimental group for MDA-MB-468) were obtained by homogenizing the tumor in 1.0 ml ice-cold Trizol (Life Technologies) using an homogenizer (Omni International) and suspending in 0.2 ml of chloroform (EMD) for 15 minutes at room temperature. Following centrifugation, the transferred supernatant was suspended in 0.5 ml of isopropanol (Fisher Scientific) for 15 minutes at room temperature to precipitate the RNA. The RNA pellet was washed in 75% ethanol and resuspended in diethylpyrocarbonate (DEPC) water (Quality Biological).

Amplification of 36B4 was used as an internal control in the qRT-PCR reaction [17]. Relative expression between samples was calculated by the comparative C_T_ method. The VEGFA primers used in this study were VEGFA_all isoforms_ (sense): 5′-CTTCCTACAGCACAACAAAT-3′, (antisense) 5′-GTCTTGCTCTATCTTTCTTTGG-3′; VEGFA_121_ (sense): 5′-ATAGAGCAAGACAAGAAAAATG-3′, (antisense) 5′-ATCGTTCTGTATCAGTCTTTCCT-3′; VEGFA_165_ (sense): 5′-AGAGCAAGACAAGAAAATCC-3′, (antisense) 5′-TACAAACAAATGCTTTCTCC-3′; VEGFA_189_ (sense): 5′-TATAAGTCCTGGAGCGTTC-3′ (antisense) 5′-TACACGTCTGCGGATCTTG -3′ and have been described previously [25]. The primers for VEGFA_all isoforms_ span exons commonly present in all isoforms of VEGFA and hence represent total VEGFA mRNA levels.

### Western blot analysis

Conditioned media from cell lines was collected by maintaining the cells in serum-free medium. Whole cell extract from tumor tissues was obtained by suspending the tumor tissue in lysis buffer composed of 20 mM Tris-HCl pH 8.0, 137 mM NaCl, 10% (w/v) glycerol, 1% Triton X-100, 2 mM Na_2_VO_4_, 2 mM EDTA and homogenizing using a homogenizer. The cell suspension was incubated on ice for 40 minutes with intermittent vortexing and centrifuged to collect the whole cell extract. Samples were resolved using SDS-PAGE, transferred to nitrocellulose membrane (Bio-Rad) and probed with rabbit anti-VEGFA (ABS82, Millipore; kindly provided by Balaji Krishnamachary, Johns Hopkins University School of Medicine, Baltimore, MD), rabbit anti-Lamin B (ab16048, Abcam), goat anti-HIF-1α (sc8711, Santa Cruz Biotechnology), and mouse anti-GAPDH (sc-47724, Santa Cruz; kindly provided by Dr. Shanmugasundaram Ganapathy Kanniappan, Johns Hopkins University, School of Medicine, Baltimore, MD) antibodies. Membranes were incubated with horseradish peroxidase-conjugated antibody against rabbit (NA934V, GE HealthCare), mouse (NA931V, GE HealthCare) or goat (NB7357, Novus Biologicals; kindly provided by Balaji Krishnamachary) IgG and binding was revealed by chemiluminescence detection (Millipore).

### Chromatin immunoprecipitation

Chromatin immunoprecipitation (ChIP) was performed on nuclear cell lysates utilizing anti-sheep CITED2 (R&D Systems) and anti-sheep IgG (Jackson ImmunoResearch) antibodies and following the SimpleChIP Enyzmatic Chromatin IP Kit (Cell Signaling Technology) protocol based on manufacturer's instructions. The promoter primer sequences used for human VEGFA were: (sense) 5′- AGACTCCACAGTGCATACGTG -3′, (antisense) 5′- AGTGTGTCCCTCTGACAATG -3′. Data are representative of two independent experiments performed in triplicate per experimental condition.

### Statistical analysis

Differences in the (1) tumor volume, percentage of deceased tissue, percentage of Ki67 positive cells, total number of blood vessels, vessel area and vessel diameter between the scramble and shCITED2-expressing MDA-MB-231 tumors, and (2) fold enrichment between CITED2 ChIP antibodies in the absence and presence of TGF-β treatment were compared by unpaired Student *t* test.

Differences in the (1) mRNA expression of VEGFA isoforms, IL-6, FGF2, ANGPT1, SEMA3C and HIF-1α in shCITED2-expressing tumors relative to scramble tumors normalized to 1.0, (2) TGF-β treated cell lines relative to untreated control normalized to 1.0 and (3) fold enrichment between CITED2 ChIP antibody and IgG ChIP antibody normalized to 1.0 in absence and presence of TGF-β treatment were compared by one sample *t* test.

A *P value* of < 0.05 was considered statistically significant. For all figures, * denotes *P* < 0.05, ** denotes *P* < 0.01, and *** denotes *P* < 0.001.

## SUPPLEMENTARY MATERIALS FIGURES AND TABLES



## Abbreviations

CITED2: Cbp/p300–interacting transactivator with Glu/Asp rich carboxy-terminal domain 2
LhX2: LIM homeobox 2 TFAP2 Transcription factor AP-2 PPARγ Peroxisome proliferator-activated receptor gamma CBP CREB binding protein VEGFA Vascular endothelial growth factor A TGF-β Transforming growth factor-beta HIF-1α Hypoxia inducible factor-1 alpha.
