# Supplementary material for: Down-regulation of CITED2 attenuates breast tumor growth, vessel formation and TGF-β-induced expression of VEGFA
Source: Oncotarget. 2016 Dec 21;8(4):6169–78. doi: 10.18632/oncotarget.14048 (PMC5351621; doi:10.18632/oncotarget.14048)
Supplement: Supplementary file 1 [file oncotarget-08-6169-s001.pdf]

Down-regulation of CITED2 attenuates breast tumor growth, vessel formation and TGF-β-induced expression of VEGFA

Supplementary Materials

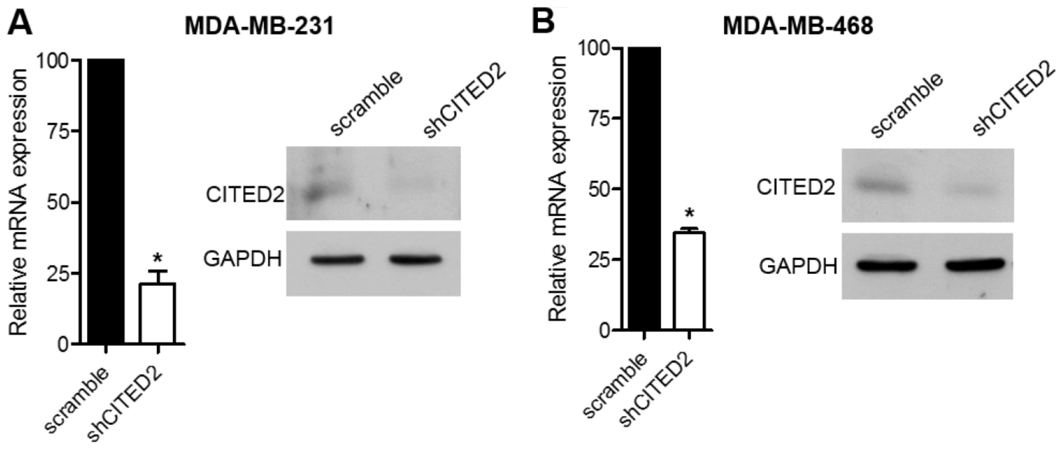

Supplementary Figure S1: Analysis of CITED2 expression in scramble and shCITED2-expressing MDA-MB-231 and MDA-MB-468 cells. (**A–B**) Left: qRT-PCR analysis of mRNA expression. Right: Western blot analysis performed on equal amounts of protein obtained from total cell lysates. GAPDH served as the loading control.

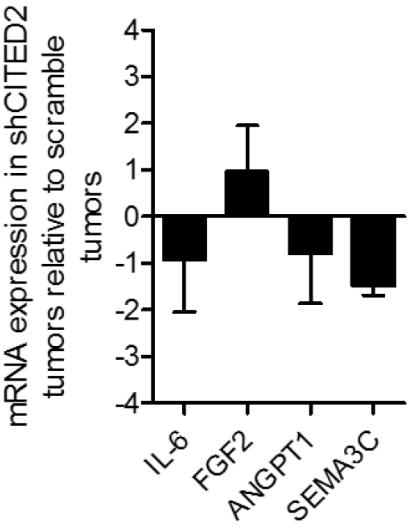

Supplementary Figure S2: CITED2 silencing does not affect the mRNA expression of pro-angiogenic IL-6, FGF2, ANGPT1 and SEMA3C in MDA-MB-231 orthotopic tumors. mRNA expression as determined by qRT-PCR analysis.

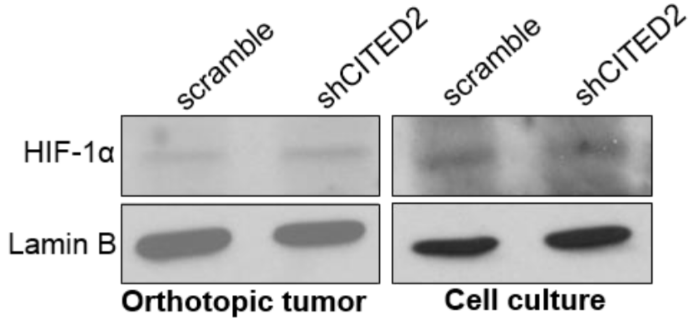

Supplementary Figure S3: CITED2 silencing does not affect the expression of HIF1α in MDA-MB-231 cells. Western blot analysis of HIF-1α expression performed on equal amounts of protein obtained from total cell lysates of orthotopic tumor (left) and cells grown *in vitro* (right). Lamin B served as the loading control.
